# Calorie information and dieting status modulate reward and control activation during the evaluation of food images

**DOI:** 10.1371/journal.pone.0204744

**Published:** 2018-11-02

**Authors:** Andrea L. Courtney, Emma K. PeConga, Dylan D. Wagner, Kristina M. Rapuano

**Affiliations:** 1 Psychological & Brain Sciences, Dartmouth College, Hanover, NH, United States of America; 2 Department of Psychology, Stanford University, Stanford, CA, United States of America; 3 Department of Psychology, The Ohio State University, Columbus, OH, United States of America; 4 Department of Psychology, Yale University, New Haven, CT, United States of America; Duke University School of Medicine, UNITED STATES

## Abstract

Several public health departments throughout North America have responded to the obesity epidemic by mandating that restaurants publish calories at the point of purchase—with the intention of encouraging healthier food decisions. To help determine whether accompanying calorie information successfully changes a food’s appetitive value, this study investigated the influence of calorie information on brain responses to food images. During functional magnetic resonance imaging (fMRI) scanning, dieting (N = 22) and non-dieting (N = 20) participants viewed pictures of food with and without calorie information and rated their desire to eat the food. When food images were paired with calorie information, not only did self-reported desire to eat the food decrease, but reward system activation (Neurosynth-defined from the term “food”) decreased and control system activation (the fronto-parietal [FP] control system) increased. Additionally, a parametric modulation of reward activation by food preferences was attenuated in the context of calorie information. Finally, whole brain multivariate pattern analysis (MVPA) revealed patterns of activation in a region of the reward system—the orbitofrontal cortex (OFC)—that were more similar for food images presented with and without calorie information in dieting than non-dieting participants, suggesting that dieters may spontaneously consider calorie information when viewing food. Taken together, these results suggest that calorie information may alter brain responses to food cues by simultaneously reducing reward system activation and increasing control system activation. Moreover, individuals with greater experience or stronger motivations to consider calorie information (i.e., dieters) may more naturally do so, as evidenced by a greater degree of representational similarity between food images with and without calorie information. Combining an awareness of calories with the motivation to control them may more effectively elicit diet-related behavior change.

## Introduction

Obesity is a worldwide public health problem, with 39% of adults categorized as overweight or obese [1]. It is associated with shortened life expectancy and higher medical care costs; the annual US medical expenditure attributable to obesity is estimated to be US $147 billion [2]. One contributing factor is that, with the abundance of available food options, consumers are not particularly good at estimating the number of calories consumed in their meals; and their estimates get worse as the size of the meal grows [3,4]. This makes it challenging to identify healthy food options and may contribute to overeating. Moreover, individual dieting histories appear to influence attention to and knowledge of various aspects of food nutrition. Dieters attend more carefully to aspects of foods that might be perceived as indicating unhealthiness, and they are more accurate at estimating the calories of healthy foods [5].

To combat a general disregard for nutrition information and disparities among consumers in using this information to inform food decisions, policy changes have been proposed to increase public awareness of the nutritional value of food alternatives for pre-packaged foods [6] and at restaurants [7,8]. Likewise, consumers around the world have voiced their preference to see these foods labeled with simple, visible nutrition information [9,10]. In one of the latest instantiations of these health policies, several U.S. public health departments have mandated that restaurants publish calories at the point of purchase to encourage healthier food decisions by consumers [11,12].

### Influence of calorie information on food choices

Regardless of its increasing presence, point-of-purchase calorie labeling has had mixed success in nudging food selection and consumption toward lower calorie options [13–17]. In fact, systematic reviews and meta-analyses targeting the influence of calorie labeling have largely yielded null or insignificantly-sized effects [18–21]. Even when it is available, only about one third of consumers report using calorie information to make food decisions [15]. Moreover, consumers over-report their use of nutrition labels; although they express an intention to use nutrition labeling, they fail to incorporate this information when making actual food decisions [9,22].

Still, a few factors appear to facilitate the influence of calorie labeling on food selection. For example, calorie labels are more effective when paired with additional interpretive labeling—like exercise equivalents [16], symbolic health icons [15,18], or recommended daily allowances [14]—which provide additional context and implications, enabling consumers to make informed decisions about their diet. Additionally, consumer characteristics appear to moderate the influence of calories on food decisions. Specifically, females [15,18] and dieters [16] tend to weigh calorie information more when making food decisions than do males and non-dieters. These person-level motivations might shape attitudes toward food and moderate the efficacy of nutrition-related interventions. To better understand the influence of calories on food valuation and choices, the present study examines their influence on brain responses during the evaluation of food cues.

### Neuroimaging studies of food reward

Food cues typically elicit robust activation in putative reward regions—including the nucleus accumbens (NAcc) and orbitofrontal cortex (OFC) (e.g., [23,24–27]), which motivate eating behavior. Individual differences in food cue reactivity relate to eating and health outcomes—including weight status [28,29], weight gain [23], post-surgery weight loss [30], body fat [25], moment to moment food cravings [24], and giving in to food cravings [31]. Although palatable foods reliably activate the brain’s reward system and motivate consumption (e.g., [23,24]), changes in valuation or enhanced regulation by cognitive control regions [32] might attenuate this response in the presence of calorie information.

### Influence of calorie information and dieting status on brain activation

Responses in reward-related brain regions, specifically the OFC, scale with idiosyncratic food preferences, such that more preferred foods elicit more reward activation [33,34]. These reward responses are also sensitive to broader contextual features, like the presence of alternative rewards [35], hunger/satiety [36], and value changes [36,37]. As most research in food reward has focused on sensitivity of the reward system to appetitive aspects of food, it is not well understood how other aspects of food, like its healthiness, may alter reward responses. One study found that the presence of health cues nudged healthier food decisions by altering value-related brain activation in the ventromedial prefrontal cortex (VMPFC) [38]. Calorie information, in particular, may signal a food’s healthiness and provide another dimension on which to evaluate food options. That is, in the absence of calorie information, people may make food decisions based on the perceived tastiness and appetitive features of a food. Conversely, when foods are presented in conjunction with calorie information, they may instead rely on their ability to self-regulate and make decisions based on health and their broader dieting goals. This shift may rely on brain-based valuation processes.

Calorie information may additionally trigger increases in cognitive control activation (e.g. the fronto-parietal [FP] system), particularly for dieters regulating food desire [32]. Prefrontal cortical activation often increases in response to food cues for populations concerned with weight management, namely those who are obese [39,40] or dieting [41]. Indeed, one study measured activation of the FP control system and the reward system in chronic dieters viewing food images [31]. Over the subsequent week, these dieters completed daily assessments of their real-world eating behaviors, including how often they gave in to a food desire. In that study, the relative balance of FP control activation to reward activation among dieters viewing food images predicted the percentage of enacted food desires over the subsequent week—highlighting the importance of these brain systems for the self-control of food desire.

Dieters may be particularly sensitive to the presence of calorie information because of their concern for restraining caloric intake. In lab studies, dieters eat fewer high-fat and unhealthy foods when their self-regulatory goals are primed by the surrounding context—e.g., by presence of a mirror [42] or thin human-like sculptures [43]. In fact, calorie labels more strongly influence dieters’ food choices [7] and how much they eat [44], even if the calorie information they receive is incorrect. Through their experience attending to food calories, dieters may learn to represent foods differently than non-dieters—possibly integrating the perceived healthiness of foods with other appetitive characteristics, even in the absence of explicit health information. By using representational similarity analysis (RSA)—which permits a more direct comparison of the shared information across different conditions [45]—we can compare dieters’ and non-dieters’ representations of food inside and outside the context of calorie information. If brain activation patterns across the two groups differentially change with exposure to calorie information, it may suggest a baseline difference in dieters’ and non-dieters’ representation of food.

Given that prior research has shown that calorie information has a strong influence on dieters’ food choices and consumption, we sought to investigate how the presence or absence of calorie information altered neural responses to food items in brain regions associated with reward and self-control. Specifically, we tested whether the presence of calorie information would simultaneously decrease reward-related activation while increasing activation in regions associated with cognitive control during the evaluation of food images. Next, we tested whether the presence of calorie information would alter the association between reward-related activation and idiosyncratic food preferences using a parametric modulation approach. Another aim of the current study was to evaluate the sensitivity of dieters’ and non-dieters’ brain responses to calorie-labeled foods. We approached this goal in three complementary ways: first, we compared activation magnitudes of as a function of dieting status; second, we compared the association between reward-related activation and food preferences by dieting status; and third, we compared differences in neural representations of calorie-labeled and calorie-unlabeled foods between these groups using representational similarity analysis.

## Materials & methods

This study was approved by the Committee for the Protection of Human Subjects at Dartmouth College (CPHS #20325).

### Participants

Fifty dieting and non-dieting participants (*M* age = 19.7, range 18–22) were recruited to participate in the present study. Participants provided their written informed consent to participate in this study. Afterward, they received monetary compensation or class credit for participating in the study. All participants reported a normal neurological history and had normal or corrected-to-normal visual acuity, and only one participant was left-handed. FMRI data were excluded for participants (n = 8) whose movement during any run of the scan exceeded 3-mm in translation or 2 degrees in rotation. Participants completed the Revised Restraint scale [46] to estimate their chronic dieting tendencies. Following convention, participants scoring greater than 14 (across both the “Weight Fluctuation” and “Concern for Dieting” subscales) were classified as dieters and those scoring 14 or lower were classified as non-dieters [47]. Dieters and non-dieters were explicitly from a participant pool based on their early Restraint scale scores, so they were treated categorically in this study. Of the 42 participants (*M* age = 19.6, 9 male) included in analyses, 22 were dieters and 20 were non-dieters. Each participant’s weight, BMI, and percent body fat (Table 1) were obtained from a Tanita body composition scale (TBF-300A Arlington Heights), which estimates body composition using bioelectrical impedance analysis. On average, participants were in the normal weight range (*M* BMI = 23.5), with only two (one dieter, one non-dieter) classifying as obese (BMI > 30).

**Table 1:** Participant characteristics by dieting status.

	Dieters (N = 22)	Non-dieters (N = 20)
Gender	16 females, 6 males	17 females, 3 males
Age (yrs)	19.2 *(1*.*1)*	20.1 *(1*.*5)*
Weight (kg)	69.0 *(9*.*8)*	65.6 *(15*.*4)*
BMI (kg/m^2^)	24.1 *(2*.*9)*	22.7 *(3*.*8)*
Body fat (%)	26.1 *(7*.*5)*	24.1 *(7*.*1)*
Restraint Scale	19.8 *(4*.*3)*	9.0 *(3*.*2)*
Weight Fluctuation	11.8 *(3*.*6)*	5.5 *(2*.*3)*
Concern for Dieting	8.0 *(2*.*6)*	3.5 *(2*.*6)*
Calorie estimation deviation	0.46 *(0*.*64)*	0.52 *(0*.*79)*

Means and standard deviations by dieting status. There were no between group differences in weight, BMI, or body fat (all *p*s > 0.18), and only a marginal group difference in age, *t*(40) = 2.03, *p* = 0.053. Dieters and non-dieters differed in both subscales of the Restraint Scale: Weight Fluctuation, *t*(40) = -6.76, p < 0.001, and Concern for Dieting, *t*(40) = -5.58, p < 0.001. Calorie estimation deviation reflects participants’ absolute proportional deviation of estimated calories from actual calories [i.e., abs(estimated calories—actual calories) / actual calories].

### Stimuli

**Fig 1:**
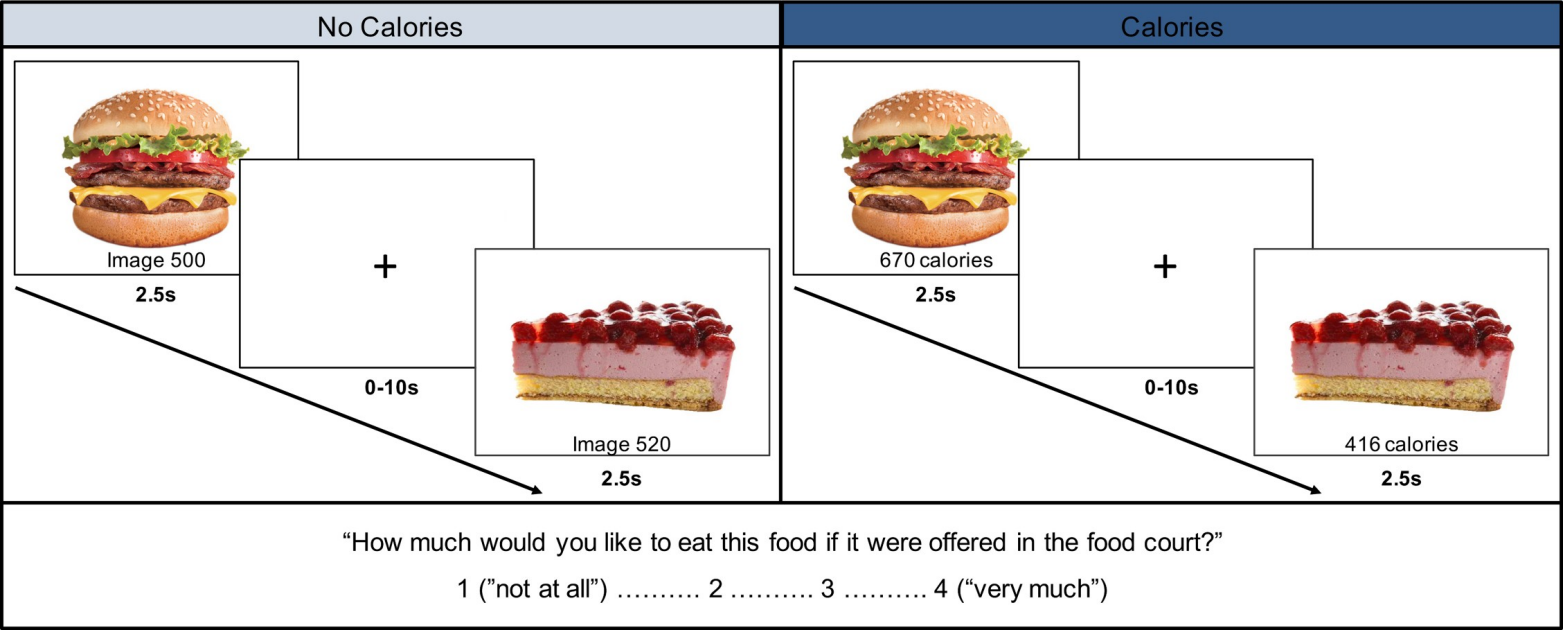
Representation of the food evaluation paradigm. 180 food images were presented (2.5s trial, 0-10s ITI) under two conditions: without calorie information and with accurate calorie information. At the beginning of each run, participants were instructed to evaluate how much they would like to eat the food presented on each trial if they saw it in the campus dining hall (1 = “not at all”– 4 = “very much”).

Stimuli consisted of 180 food images collected from two sources: 1) the *food-pics* database [48], and 2) from popular fast food restaurant websites where calorie information (total kilocalories) was provided. Images selected from the internet were edited to replace any existing backgrounds with a white background and were resized to 650 x 450 pixels using Adobe Photoshop. As part of the manipulation of interest, each food image was paired with either an arbitrary number (e.g., “image 002”) or calorie content (e.g., “450 calories) associated with the food—which appeared on the screen below the food image (Fig 1). The same images were displayed twice—once with the image number and once with accurate calorie information (*M* = 396.5, *SD* = 240.2).

### Experimental design and procedure

During the scan, participants first viewed the images of food paired with an accompanying image number (NO CALORIES: runs 1–2), and subsequently viewed these same food images paired with the corresponding calorie information (CALORIES: runs 3–4) (Fig 1). Under the guise of gauging college student food preferences for the dining hall, participants were asked to indicate how much they would like to eat each of the foods if it were offered in the campus dining hall on a scale from 1 to 4 (1 = “not at all”, 4 = “very much”). In this manner, we obtained an explicit food preference for each item while ensuring that the participants were paying attention to the images. On trials when calorie information was presented, they were told that because federal regulation might require eating establishments to provide nutritional information for their foods, we were interested in how it might influence food choices and preferences. So that participants were not thinking about calories when none were present, all calorie-present trials came after the calorie-absent trials.

Following the scan, participants again viewed all of the same food images and were asked to estimate the number of calories for each food. To obtain true estimates of food calories, rather than relying on participants’ memory for the previously presented calorie information, we falsely informed participants that the calories they had observed during the task may have been altered for the study (and could be inaccurate) and should be disregarded when making their estimates.

### Behavioral data analysis

To analyze the difference in behavioral food ratings across condition (CALORIES/NO CALORIES), a linear mixed-effects model was used with the lme4 package [49] in R [50]. Random intercepts for both subject and item (food image) were entered into the model to account for variability in ratings attributable to the particular food or person-level preferences. Pooled degrees of freedom using the Satterthwaite approximation are reported for each effect.

### Imaging apparatus

Imaging was performed on a Philips Intera Achieva 3.0 Tesla scanner (Phillips Medical Systems, Bothell, WA) using a 32-channel SENSE (SENSitivity Encoding) head coil. Stimuli were presented from an Apple MacBook Pro laptop computer running PsychoPy v1.80 software [51]. An Epson (model ELP-7000) LCD projector displayed the stimuli on a screen at the head end of the scanner bore. Subjects viewed that screen through a mirror mounted on top of the head coil. An MR compatible keypress interfacing with the Cedrus Lumina Box recorded participant’s responses.

### Image acquisition

Anatomical images were acquired using a high-resolution 3-D magnetization-prepared rapid gradient-echo sequence (TR = 9.9ms; TE = 4.6 ms; flip angle = 8°; 1x1x1mm^3^ voxels). Functional images were collected using T2* fast field echo, functional EPIs sensitive to BOLD contrast (TR = 2.5 seconds, TE = 35 ms, flip angle = 90°, 3x3x3mm^3^ voxels). During each of the four functional runs, 142 axial images (35 slices) were acquired allowing complete brain coverage. The presentation sequence of food images and jittered fixation trials (inter-trial interval of 0-10s; 30% of total duration), were pseudo-randomized to increase the efficiency of estimating task-related BOLD activation.

### Image preprocessing

Neuroimaging data were analyzed using SPM8 (Wellcome Department of Cognitive Neurology, London, UK) in conjunction with a suite of preprocessing and analysis tools (https://github.com/ddwagner/SPM8w). The functional data were slice time corrected, realigned within and across runs to correct for head movement and transformed into a standard anatomic space (3-mm isotropic voxels) based on the ICBM 152 brain template space [Montreal Neurological Institute (MNI)]. Normalized data were then smoothed spatially using a 6-mm Gaussian kernel. To further account for motion artifact, participants that demonstrated substantial movement (> 3-mm in translation or 2 degrees in rotation) were discarded.

### Whole brain comparison of food images > calorie-labeled food images

For each participant, a general linear model (GLM) incorporating task effects (modeled as onsets of events of interest convolved with the canonical hemodynamic response function), were used to compute beta images (parameter estimates) representing task-related effects for each voxel in the brain. Nuisance regressors included six motion parameters (x, y, z directions and roll, pitch, yaw rotations), a linear drift, and run constants. The resulting beta images were used to compute a whole-brain voxelwise contrast comparing the food images to the calorie-labeled food images. Subsequent contrast maps were used as input into a group-level random-effects analysis to identify brain areas that were consistently active across participants. The resulting group-level map was voxelwise thresholded at p < 0.005, and cluster corrected to p < 0.005 (minimum extent threshold: k = 148 contiguous voxels) using AFNI’s 3dClustSim with the spatial autocorrelation function to control false positive rates (see [52,53]). The minimum cluster size required for whole-brain multiple comparisons correction was calculated from Monte Carlo simulations (10,000 iterations).

### Region of interest (ROI) analyses

Because research on the self-regulation of food reward has pointed to the opposing influence of reward and cognitive control systems on food decisions [32,38,62], we predicted that calorie information might modulate both of these systems. To address these predictions, we extracted parameter estimates from both brain systems by applying two respective ROI masks. To examine reward system activation to food cues, we defined an ROI mask from a large-scale online meta-analytic search for the term “food” (reverse inference, p < 0.01 [54]). The activation map was downloaded from *neurosynth.org* and converted to a binary inclusion mask to target activation associated with food reward. This approach yielded a spatially constrained set of cortical and subcortical regions that were more likely to appear in studies related to food than those that unrelated to food, including the left OFC and the NAcc. The cognitive control system mask was assessed by averaging activation across eight nodes within the fronto-parietal control system ([55,56]; nodes listed in Table 2). To create this mask, an 8-mm sphere was centered over each of the eight nodes, and parameter estimates were extracted and averaged across all eight regions to derive a single composite measure of FP control activation for each participant. Linear mixed-effects models with random intercepts for subjects, to account for individual differences in the recruitment of brain regions when viewing food images, were used to compare neural responses in these ROIs during food evaluations across condition and dieting status.

**Table 2:** Table of eight fronto-parietal control system nodes used in *a priori* analysis [55,56]. An 8-mm sphere was centered over each node and parameter estimates averaged across regions to calculate the aggregate mean FP activation for each participant.

Regions	Coordinates (MNI)		
	X	Y	Z
R dorsolateral prefrontal cortex / inferior frontal gyrus	46	28	31
L dorsolateral prefrontal cortex / middle frontal gyrus	-44	27	33
R middle frontal gyrus / inferior frontal gyrus	44	8	34
L middle frontal gyrus / precentral gyrus	-42	7	36
R inferior parietal lobule / supramarginal gyrus	54	-44	43
L inferior parietal lobule	-53	-50	39
R intraparietal sulcus / angular gyrus	32	-59	41
L intraparietal sulcus / inferior parietal lobule	-32	-58	46

### Parametric modulation by food ratings

To compare the relative contribution of whole-brain activations to food ratings across the two conditions, a parametric modulation analysis was conducted. Using the food image ratings (1–4) collected during the scan, a subject-level regressor was entered into a first-level GLM to identify brain regions whose activity increased linearly with increasing food preference ratings. One regressor corresponding to the food preference rating was entered for each subject at each timepoint. Specifically, though each food was presented twice (once per condition), food preference ratings were collected both times; and brain activation was regressed with the ratings acquired during the same trial. Next, a second-level t-test was conducted to compare parametric modulation for the two conditions (NO CALORIES > CALORIES). The resulting group-level map was voxelwise thresholded at p < 0.005, and cluster corrected to p < 0.005 (minimum extent threshold: k = 38 contiguous voxels) as recommended by AFNI’s 3dClustSim with the spatial autocorrelation function (see [52,53]). Parameter estimates were extracted from the reward system (Neurosynth-defined “food” mask) ROI to determine whether the association between reward activation and food preferences [33] were attenuated in the presence of calorie information.

### Representational similarity analysis

A whole brain searchlight (radius = 3 voxels) representational similarity analysis (RSA; [45,57]) using multivariate pattern analysis (MVPA) with the PyMVPA software package [58] was conducted to compare the similarity in activation patterns across the CALORIE and NO CALORIE conditions. Voxel-level Fisher-z transformed correlation values (1 –correlation distance) representing similarity across the CALORIE and NO CALORIE conditions were submitted to a group t-test comparing dieters and non-dieters to identify regions where dieters more similarly represented foods regardless of condition (CALORIES/NO CALORIES) than non-dieters. The resulting group-level map was voxelwise thresholded at p < 0.01. Though no clusters survived, the minimum extent threshold required for whole-brain cluster correction to p < 0.01 was 459 contiguous voxels.

## Results

### Behavioral results

**Fig 2:**
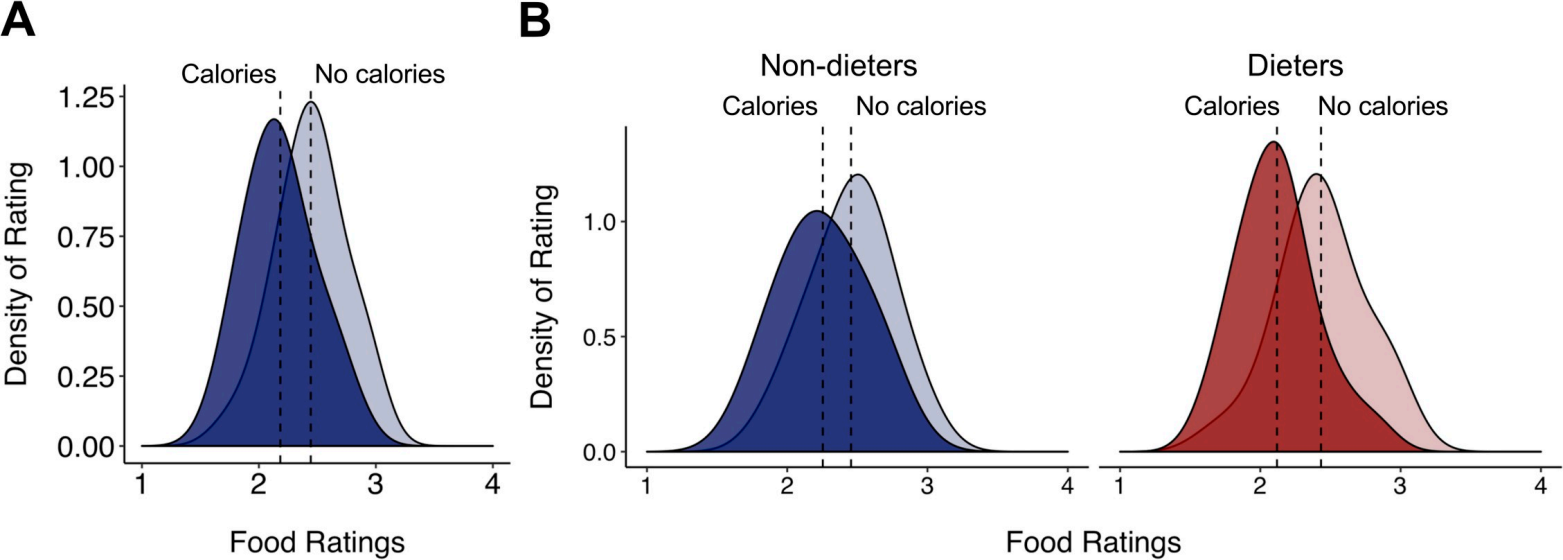
(A) Ratings of willingness to eat foods presented in images with NO CALORIES and with CALORIES. Willingness to eat was lower when presented with calories—and this difference was greater for dieters than non-dieters. (B) Food ratings by dieting status.

Food ratings decreased when images were paired with calorie information than when paired with an arbitrary image number, *B* = 0.13, 95% CI [0.12, 0.15], *t*(14180) = 17.24, p < 0.001 (Fig 2A). A second model tested a difference in behavioral food ratings across condition and dieting status (dieters vs. non-dieters), with a random intercept for subject and item (to control for individual preferences and variations in baseline ratings of individual foods, respectively). Overall, the reduction in food ratings when calories were present was stronger for dieters than non-dieters, *B* = 0.06, 95% CI [0.03, 0.09], *t*(14180) = 3.71; p = 0.0002 (Fig 2B). The absolute proportional deviation in calorie estimates from actual calories deviation [i.e., abs(estimated calories—actual calories) / actual calories] did not differ for dieters and non-dieters, *B* = -0.06, 95% CI [-0.14, 0.01], *t*(40) = 1.66; p = 0.10.

### fMRI results

**Fig 3:**
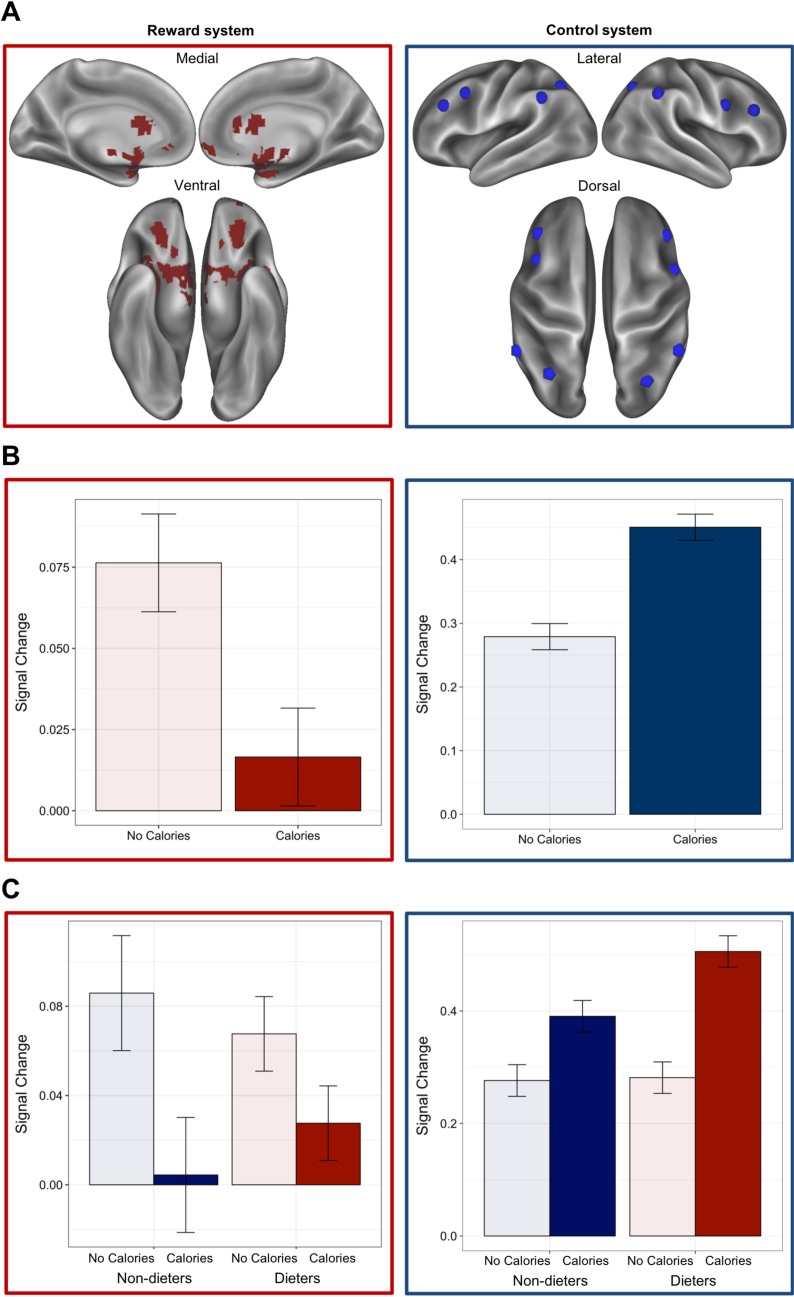
(A) ROI masks of the reward system (meta-analytic map of “food” [54]) and fronto-parietal control system [55,56]. (B) Greater activation in the reward system for NO CALORIES > CALORIES, and greater activation in the fronto-parietal control system for CALORIES > NO CALORIES. (C) No difference in non-dieters’ and dieters’ recruitment of the reward system but marginally significant difference in recruitment of control system when evaluating calorie-labeled and unlabeled foods. Dieters more strongly activated the fronto-parietal control system in response to calorie-labeled food images. Error bars represent standard error of the mean.

There was a system (reward vs. FP control) by condition (CALORIES vs. NO CALORIES) interaction, *B* = -0.22, 95% CI [-0.31, -0.14], *t*(123) = -5.19, *p <* 0.001, with calorie-labeled food images eliciting less reward and more control activation. Compared to food images presented without calories, those presented with calories elicited less reward activation in the *a priori* mask defined from a meta-analysis of the term “food” ([54]; Fig 3), *B* = 0.08, 95% CI [0.02, 0.14], *t*(40) = 2.64, *p* = 0.01. A similar relationship was found in an *a priori* defined region of the OFC (S2 Fig). In contrast, activation in the FP control system was greater for food images paired with calorie information, *B* = 0.11, 95% CI [-0.003, 0.19], *t*(40) = 2.80, *p* = 0.008. Moreover, there was a marginally significant interaction of condition (CALORIES vs. NO CALORIES) and dieting status (DIETERS vs NON-DIETERS) on the FP control system, *B* = 0.11, 95% CI [-0.00003, 0.22], *t*(40) = 1.96, *p* = 0.06, but not the reward system, *B* = -0.04, 95% CI [-0.12, 0.04], *t*(40) = -0.97, *p* = 0.34. Dieters showed a greater increase in FP control activation in the context of calories, *B* = 0.22, 95% CI [0.15, 0.30], *t*(21) = 5.67, *p* < 0.001, than non-dieters, *B* = 0.11, 95% CI [0.03, 0.19], *t*(19) = 2.87, *p* = 0.01.

**Fig 4:**
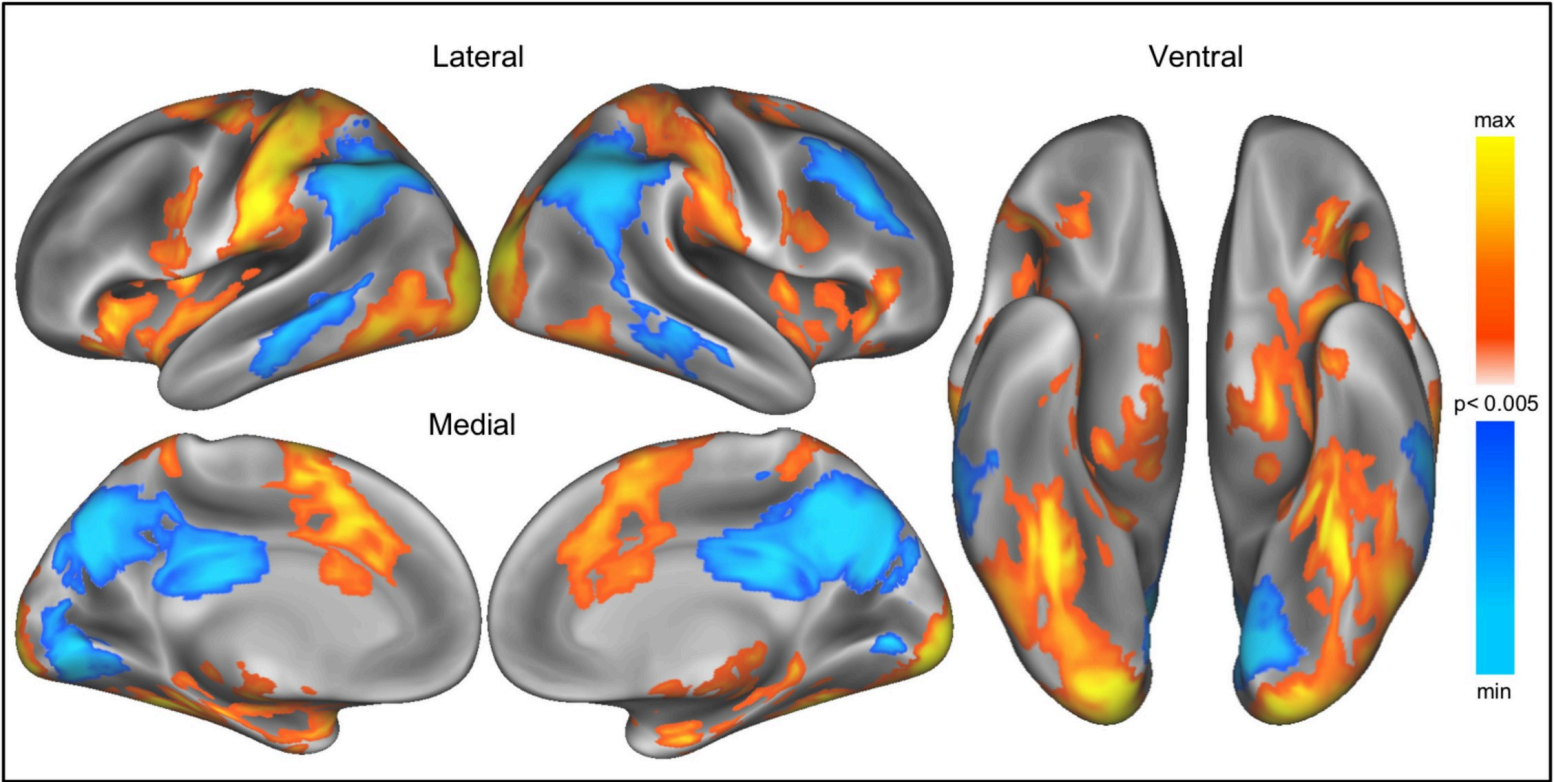
Brain regions showing greater activation to food presented with NO CALORIES > CALORIES. Whole brain cortical activations (voxelwise p < 0.005, cluster-corrected to p < 0.005, 148 contiguous voxels) are projected onto a 3D rendering of an inflated brain using Workbench [59,60] for data visualization. Brain regions responding to NO CALORIES > CALORIES (represented by yellow-red color scale) include the left middle occipital gyrus, left supramarginal gyrus, right postcentral gyrus, right inferior frontal gyrus, and right insula, and brain regions responding to CALORIES > NO CALORIES (represented by blue color scale) include the right inferior parietal lobule, left inferior parietal lobule, right precuneus, left lingual gyrus, right middle frontal gyrus, and right middle temporal cortex.

Additional peak activations from the whole-brain contrast of NO CALORIES > CALORIES (voxelwise p < 0.005, cluster-corrected to p < 0.005; Fig 4) were identified in the left middle occipital gyrus, left supramarginal gyrus, right postcentral gyrus, right inferior frontal gyrus, and right insula (Table 3A); activations associated with CALORIES > NO CALORIES were identified in the right inferior parietal lobule, left inferior parietal lobule, right precuneus, left lingual gyrus, right middle frontal gyrus, and right middle temporal cortex (Table 3B). Additional sub-cluster peaks for the contrast of NO CALORIES > CALORIES appeared in regions of the reward system, including the left OFC (-30, 27, -6: *t* = 5.44), left NAcc (-9, 3, -6: *t* = 4.15) and right NAcc (6, 3, -6: *t* = 3.57).

**Table 3:** A) Regions activating to NO CALORIES > CALORIES (voxelwise p < 0.005, cluster-corrected to p < 0.005, 148 contiguous voxels). B) Regions activating to CALORIES > NO CALORIES (voxelwise p < 0.005, cluster-corrected to p < 0.005, 148 contiguous voxels).

Region	Coordinates (MNI)			Volume (mm^3^)	Peak T
	X	Y	Z		
NO CALORIES > CALORIES					
Left middle occipital gyrus	-33	-87	6	6594	9.78
Left supramarginal gyrus	-60	-21	33	3130	7.58
Right postcentral gyrus	66	-18	36	923	5.65
Right inferior frontal gyrus	54	36	3	193	5.14
Right insula	42	-3	9	181	4.67
CALORIES > NO CALORIES					
Right inferior parietal lobule	39	-51	48	1481	8.86
Left inferior parietal lobule	-54	-57	51	1608	8.76
Right precuneus	6	-69	39	1371	8.70
Left lingual gyrus	-6	-81	-6	371	8.55
Right middle frontal gyrus	45	27	33	382	6.68
Right middle temporal cortex	69	-45	-6	203	5.03

Volumes refer to entire supra-threshold clusters.

Cluster peaks are indicated by their region names (adapted from Automated Anatomical Labeling in SPM).

### Reward activation linearly increased with food preferences for foods presented without calories

**Fig 5:**
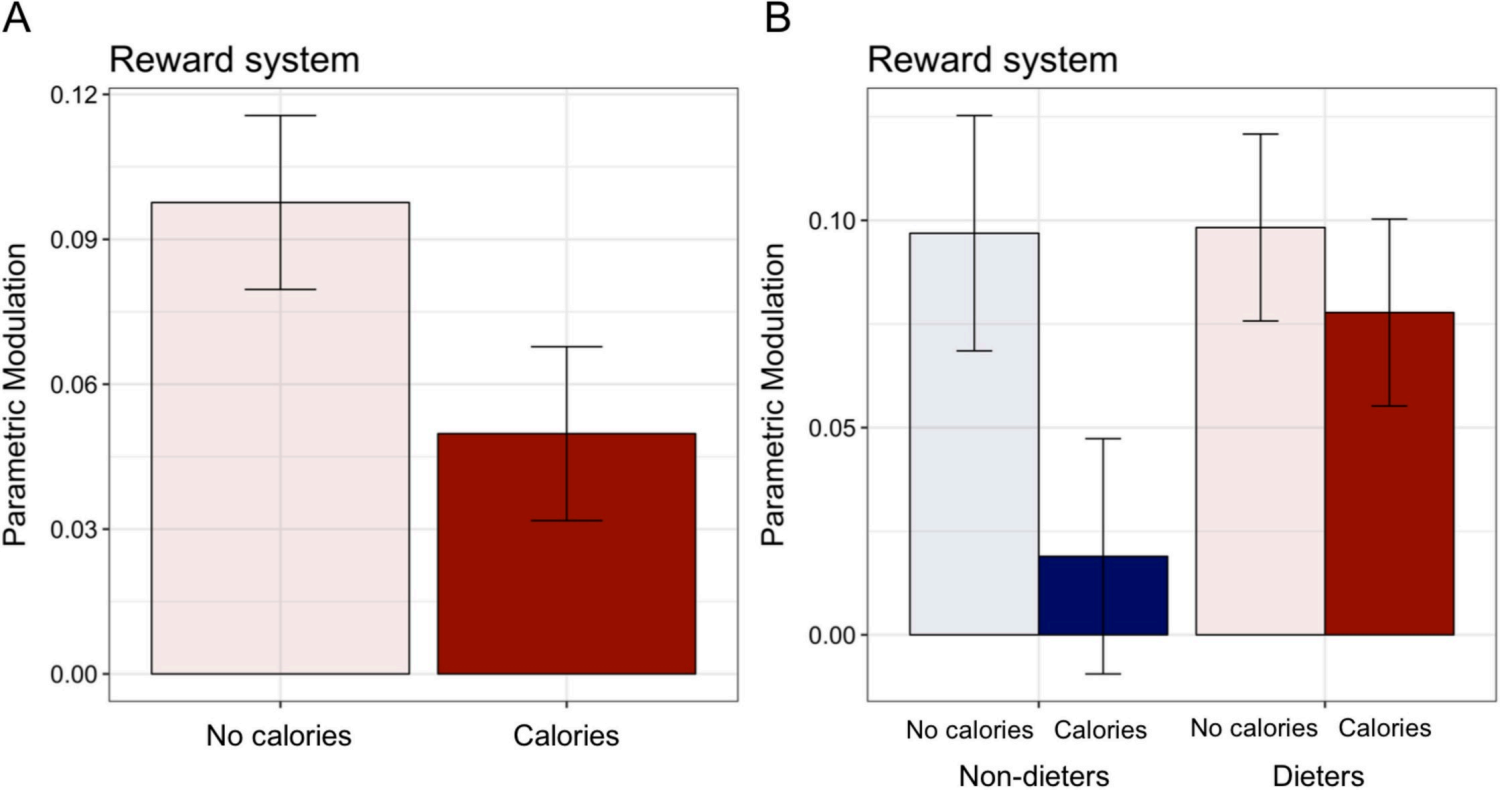
Parametric modulation by food preferences in the reward system. A) There was a stronger positive association between reward system activation and food preferences when evaluating food images with NO CALORIES than when evaluating food images with CALORIES in the reward system, *B* = 0.08, 95% CI [0.006, 0.15], *t*(40) = 2.12, *p* = 0.04. B) This relationship did not differ for dieters and non-dieters, *B* = -0.06, 95% CI [-0.16, 0.04], *t*(40) = -1.13, *p* = 0.26.

A parametric modulation analysis revealed a stronger positive relationship between activation of the reward system and food preferences when evaluating food images with NO CALORIES than when evaluating food images with CALORIES in the reward system, *B* = 0.08, 95% CI [0.006, 0.15], *t*(40) = 2.12, *p* = 0.04 (Fig 5). This relationship did not differ for dieters and non-dieters, *B* = -0.06, 95% CI [-0.16, 0.04], *t*(40) = -1.13, *p* = 0.26. A similar relationship was found in an *a priori* defined region of the OFC (S1 Fig).

**Fig 6:**
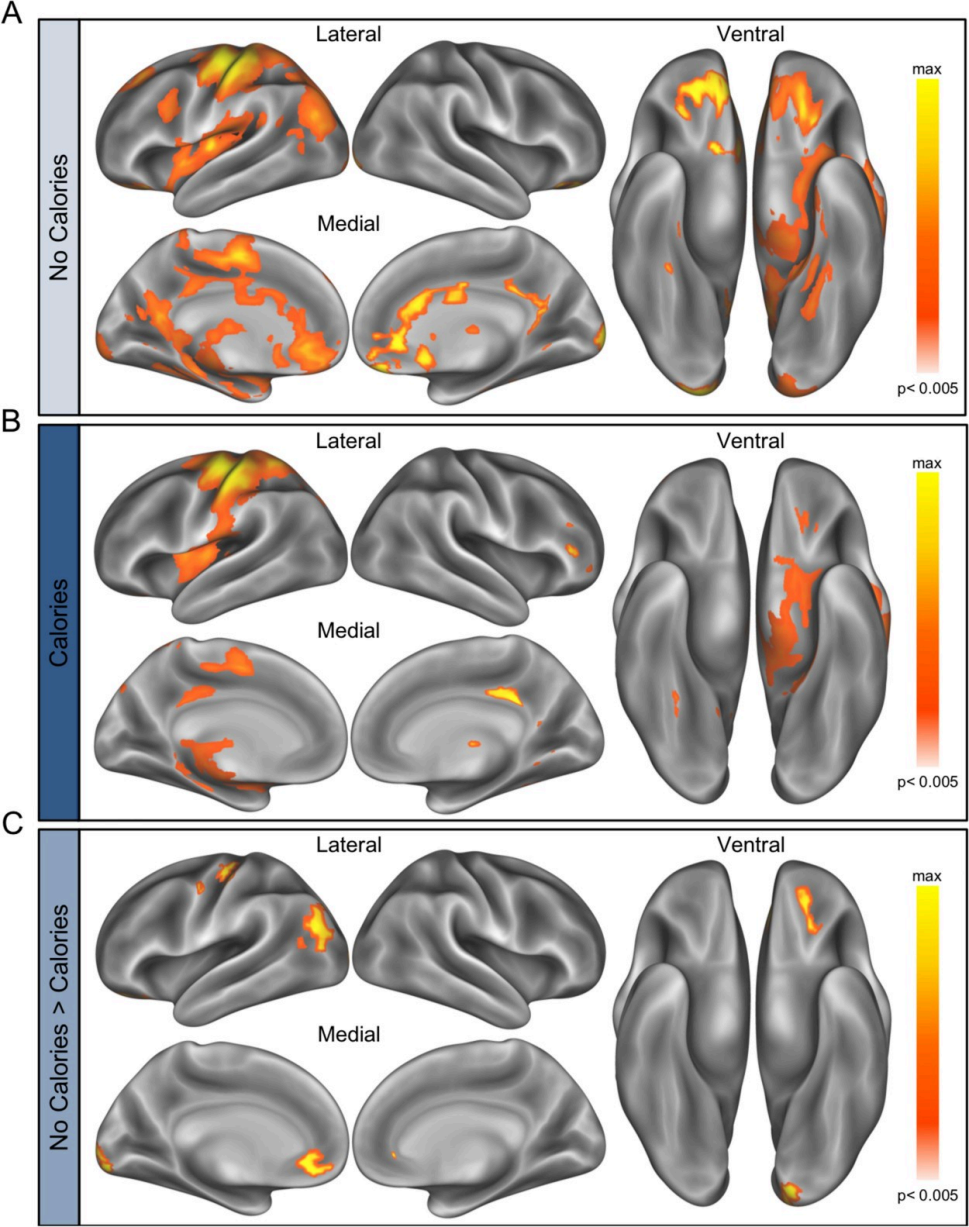
Parametric modulation by food preferences in the whole brain. (A) Brain regions whose activation when evaluating food images presented with NO CALORIES linearly increased with food preference ratings (voxelwise p < 0.005, cluster-corrected to p < 0.005, 114 contiguous voxels). (B) Brain regions whose activation when evaluating food images presented with CALORIES linearly increased with food preference ratings (voxelwise p < 0.005, cluster-corrected to p < 0.005, 127 contiguous voxels). (C) Greater association between brain activation and food preferences when evaluating food images with NO CALORIES than when evaluating food images with CALORIES in the left OFC, left cuneus, left medial OFC, left occipitotemporal cortex, and left precentral gyrus (voxelwise p < 0.005, cluster-corrected to p < 0.005, 38 contiguous voxels).

**Fig 7:**
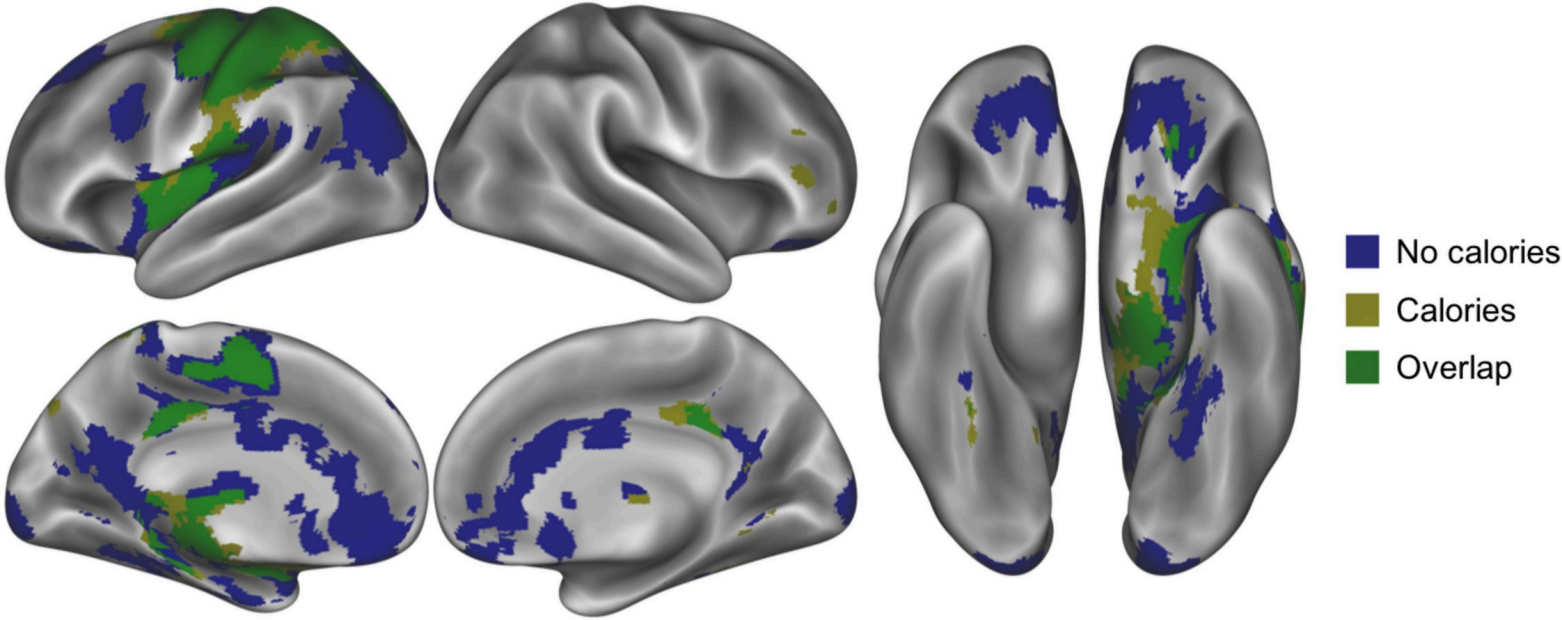
Parametric modulation by food preferences for NO CALORIES and CALORIES. Regions whose activation linearly increased with food preferences when NO CALORIES were present (but not when calories were present) depicted in blue. Regions whose activation linearly increased with food preferences when CALORIES were present (but not when calories were absent) depicted in yellow. Overlapping regions whose activation linearly increased with food preferences for both conditions depicted in green.

Several additional brain regions revealed a stronger positive relationship between brain activation and food preferences when evaluating food images with NO CALORIES than when evaluating food images with CALORIES, including left OFC, left cuneus, left medial OFC, left occipitotemporal cortex, and left precentral gyrus (voxelwise p < 0.005, cluster-corrected to p < 0.005; Table 4, Fig 6). Importantly, though this analysis was exploratory in nature, food ratings were more robustly associated with activation in regions of the left OFC (-24, 45, -18) and left medial OFC (-6, 42, -12) in the absence of calorie information. Though these regions have previously been associated with tracking food preferences [33,34,38], here we demonstrate that this relationship is attenuated in the context of additional calorie information. Additional correspondence between precentral gyrus activation and food ratings might reflect overall differences in the ratings—and associated button presses—of unlabeled and calorie-labeled foods. Widespread brain activation, including within regions involved in reward and valuation, scaled with the desirability of the foods, but this relationship was largely was diminished in the presence of calorie information (Fig 7).

**Table 4:** Brain regions whose responses were more related to food preferences when evaluating food images with NO CALORIES than when evaluating food images with CALORIES (voxelwise p < 0.005, cluster-corrected to p < 0.005, 38 contiguous voxels).

Region	Coordinates (MNI)			Volume (mm^3^)	Peak T
	X	Y	Z		
Left orbitofrontal cortex / Left middle frontal orbital gyrus	-24	45	-18	61	4.78
Left cuneus / Left middle occipital gyrus	-18	-99	6	99	4.78
Left medial orbitofrontal cortex	-6	42	-12	68	4.68
Left occipitotemporal cortex / Left middle occipital gyrus	-30	-84	39	146	4.52
Left precentral gyrus	-30	-27	54	94	3.80

Volumes refer to entire supra-threshold clusters.

Cluster peaks are indicated by their region names (adapted from Automated Anatomical Labeling in SPM).

### Multivariate activation patterns across conditions differed more for dieters than non-dieters

**Fig 8:**
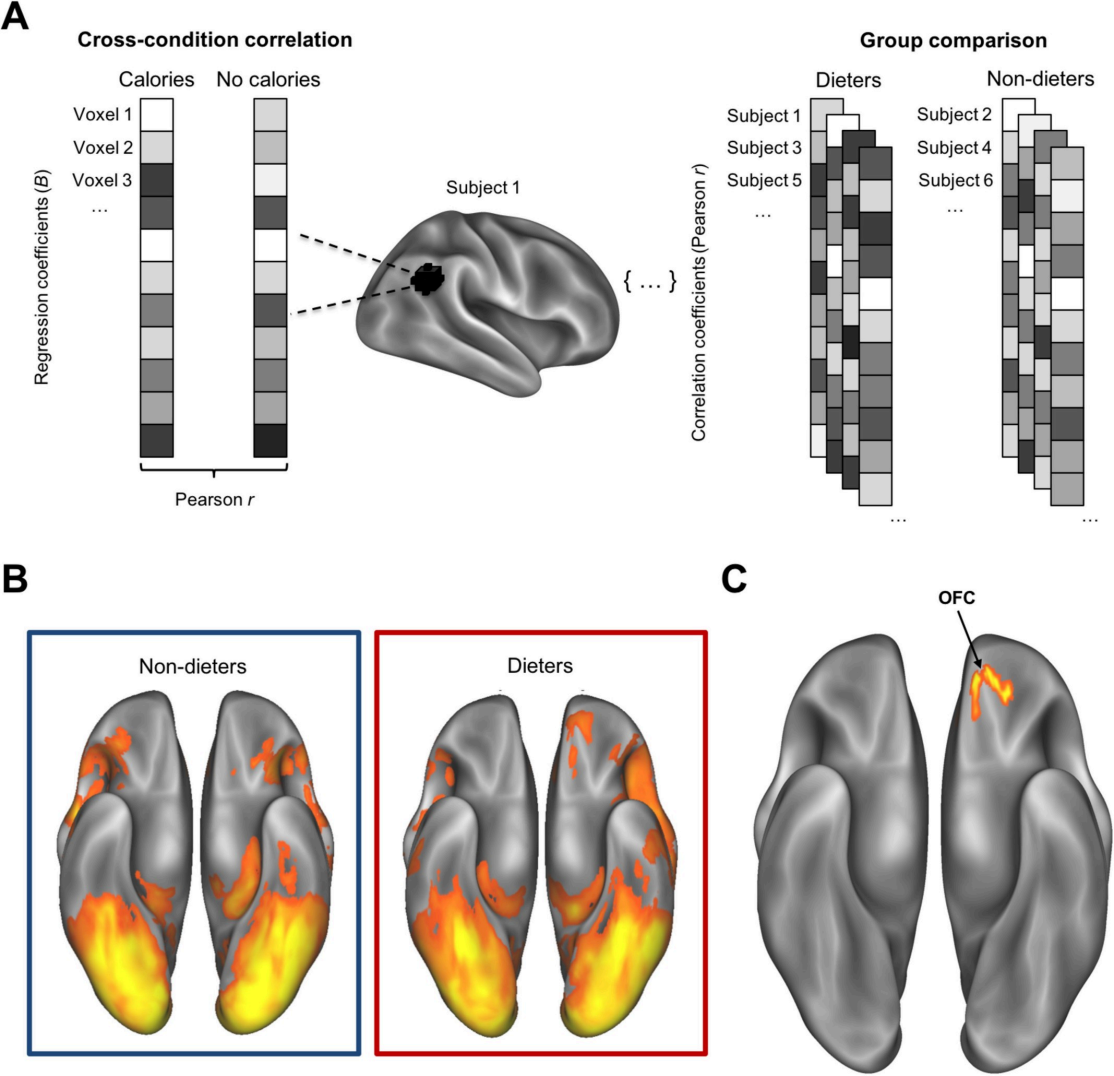
(A) Schematic of multivariate searchlight RSA comparing activation to foods images presented with and without calories. All voxels within a 9-mm searchlight sphere contribute to the cross-condition correlation value at that location. This process is iterated across the entire brain to generate a whole-brain cross-condition correlation map for each subject. Between subjects, a whole-brain group comparison reveals any group differences in cross-condition correlation values. (B) Statistical maps of whole-brain cross-condition correlations for non-dieters and dieters (voxelwise t > 10, cluster correction p < 0.001). (C) Statistical map representing the interaction between group (DIETERS > NON-DIETERS) and condition (NO CALORIES/CALORIES) pattern similarity from multivariate searchlight RSA (voxelwise p < 0.01, thresholded to 60 contiguous voxels), featuring one cluster in the left OFC (-18, 42, -15, ventral view).

A whole-brain searchlight RSA was performed to identify regions of the brain whose activation patterns were most similar between conditions (CALORIES/NO CALORIES; Fig 8). A group t-test compared dieters to non-dieters to identify regions where dieters showed more similar activation patterns for food images presented with and without calories. Though no clusters survived the cluster-correction threshold recommended by 3dClustSim, at a voxelwise threshold of p < 0.01, the largest cluster (67 contiguous voxels) was in the left OFC (-18, 42, -15).

## Discussion

Overall, we found that calorie information altered food evaluations and brain responses during the evaluation of food images, and that responses to calorie information differed between dieters and non-dieters. In the present study, calorie-labeled foods were rated less appetizing than foods presented without calories, and this difference was larger for dieters than non-dieters. Moreover, the presence of calorie information was associated with decreased activation of a reward system defined from a meta-analysis of task-based studies on food and increased activation in the FP control system. Interestingly, results from the parametric modulation analysis replicated previous research [33] in demonstrating that reward system responses in the left OFC linearly increased with food preferences—but in this study a weaker association was observed when calorie information was present. These results suggest that the reward system and self-reported food desire may be most sensitive to the perceived tastiness of the foods when no calories are present—but when calories are present, a second, competing source of information (healthiness) influences the valuation and ratings of foods in a more restricted OFC region of the system.

Although Hare and colleagues [38] found that the VMPFC tracked with both the healthiness and tastiness of foods and influenced food decisions, we found a weaker association between reward system activation and self-reported food desire in the context of calorie information, suggesting that under normal conditions, taste may govern the value and desirability of foods. By contrast, health-related features of foods may become more salient when cued externally (e.g., with calorie labeling) or when it is a personally-relevant feature of the food (i.e., for dieters). In fact, Hare and colleagues [38] demonstrated a bias towards healthier food decisions when participants explicitly evaluated the healthiness of foods, however, no such bias occurred when evaluating the tastiness of foods. In the absence of explicit task instruction, participants tended to highly weight perceived tastiness when making food decisions.

Another novel aspect of the present study compared activation patterns for calorie-labeled and unlabeled food across dieters and non-dieters. Dieters often show altered reward and control responses to food cues [31, 61]—and they are more attentive to and better at estimating calories than non-dieters [5]. Here, it was expected that dieters and non-dieters might respond differently to calorie-labeled food. Whereas the univariate analyses conducted here were useful in elucidating magnitude differences in brain responses, multivariate pattern analysis (MVPA) is more sensitive to the representational content of the information within an activated region [45,57] and may be a more useful source of information about higher-level semantic similarities between conditions. We compared patterns of activation associated with each condition to determine whether the cross-condition similarity differed between dieters and non-dieters. In fact, dieters’ activation patterns were more highly correlated across conditions in a region of the left OFC frequently implicated in food reward [25,27,33], suggesting a more similar representation of calorie-labeled and unlabeled food. One interpretation of this finding is that dieters may spontaneously represent and consider calories (or some marker of a food’s healthiness) when viewing food, even in their absence. This is supported by previous research demonstrating the ability of explicit health cues to influence healthier food decisions in non-dieters, a relationship that is additionally reflected by increased valuative processing in the VMPFC [38]. Additionally, non-dieters in that study demonstrated increased functional coupling between the VMPFC and the DLPFC—a region associated with self-control—mimicking a regulatory strategy employed by dieters when exerting self-control over food desires [62]. As no clusters survived correction in the present study (p < 0.01, minimum extent threshold: k = 459 contiguous voxels recommended by 3dClustSim) for the group comparison, future studies should replicate and strengthen this effect by collecting data from larger samples of dieters and non-dieters.

Additionally, considering that 79% of participants in the current sample were female, this work could be further strengthened with a more gender-balanced replication. Previous work has demonstrated that females are more responsive to calorie information when making food decisions than males [15,18]. If so, the brain responses to calorie information seen in the present study may not generalize as strongly to a predominantly male population either. Moreover, beyond the mere presence of calorie information, idiosyncrasies in calorie estimation ability might shape brain and behavioral responses to calorie-labeled food. To ensure the broadest impact, future health interventions and policies should consider the motivational, individual, and societal factors that moderate access and attention to nutritional information and food options. Consumer subpopulations have differential access to food alternatives, health care, and nutrition information, as well as different motivations for food selection (e.g., weight management, meal price, or feeding multiple family members). All of these factors contribute to decisions about food and should be used to inform policy related to food choice and availability.

One important limitation of the current study was that food images were always presented first without calorie information. This was a purposeful and necessary part of the study paradigm; the alternative—presenting some food images with calorie information first—would have primed participants to implicitly consider calorie information for subsequently presented food images. As such, reduced reward system activation in isolation, could be attributed to run order effects. Several factors, however, mitigate this potential confound somewhat. First, activation in the FP control system increased from earlier to later runs, suggesting that whole-brain habituation to the stimuli cannot account for the reductions in reward activity to foods presented with calories. Second, the multivariate analysis—which reflects differences in the representation of foods across conditions for dieters and non-dieters—bolsters our confidence that magnitude differences in reward activity are not driven by habituation effects. In RSA, activation patterns are correlated across the two conditions, specifically normalizing any (potentially spurious) magnitude-level differences. Moreover, the presence of a group difference (dieters vs. non-dieters) in pattern similarity points to meaningful individual differences in the processing of calorie-labeled and unlabeled food, which may be further influenced by person-level motivations toward food.

On a broader level, these results speak to the sensitivity of reward responses to contextual health information. In fact, many of the exploratory analyses corroborated the results of our planned analyses and predictions: calorie-labeled food elicited a differential response in the reward system. Both the whole-brain analysis of activation modulation by food ratings and cross-condition similarity in activation patterns revealed, among others, differences in a region of the left OFC. This same region has been implicated in supporting reward responsivity to food [25,33,34] and alcohol cues [63] across many studies, with individual differences in its activation relating to domain-specific consumption and health outcomes. In the present study, we find similar activation of the OFC to food cues, but this activation and its association with idiosyncratic food preferences is attenuated in the presence of calorie information. Moreover, activation of the OFC by food cues may differ based on dieting status. That is to say that behaviorally-relevant reward processing in the OFC may be sensitive less appetitive aspects of food (e.g., healthiness) when attention to these aspects is explicitly engaged (by external cues) or motivationally relevant (for dieters).

Future public policy work may consider leveraging the sensitivity of the reward system to contextual information about potential reward options when mandating product labeling. Indeed, recent research on smoking cue reactivity demonstrated that graphic warning labels successfully altered smoking cravings and neural reactivity to smoking cues [64,65], as well as the subjective value of cigarettes [66]—but these messages may be more persuasive for light smokers than regular smokers [64,67]. Considered in the context of the present results, these findings might suggest a brain-based mechanism—through altered responses in reward and control systems of the brain—by which health policies can effectively address these public health crises and elicit behavior change. However, from the present results, we cannot conclude anything about the long-term efficacy of these interventions in changing health-related behavior. In fact, one study observed a waning efficacy of calorie labeling at fast-food restaurants over a five year period—as consumers habituated to the labels their visits to fast-food restaurants and number of calories purchased returned to baseline [68]. This poses an inherent challenge for selecting a lasting intervention, which may be addressed by considering the unique motivations of various consumer subpopulations.

Interestingly, in this study, calorie information induced a greater change in the self-reported food preferences of dieters relative to non-dieters, yet non-dieters evoked a greater change in the reward-related neural representation of foods when calorie information was presented. These findings may reflect trait level differences in motivation and attention to calorie information at different levels. In fact, counteractive control theory suggests that exposure to unhealthy, tempting foods might increase self-control among dieters by increasing the value of their long-term goals for weight management [69]. Dieters might automatically consider the healthiness of foods in such a way that allows their mental representations of food to be less affected by external cues such as calorie information; however, the explicit reminder of calorie information may trigger stronger motivational control over behavior in dieters than in non-dieters. This increase in control might explain the decrease in desirability ratings for foods in the current study after the calorie content was revealed. Conversely, in a previous study, non-dieters exposed to tempting olfactory food cues—the smell of baking cookies—reported an increased importance of dieting when compared to non-dieters that were not exposed to food cues, however, the same manipulation did not influence the self-reported importance of dieting among dieters [70]. Our neuroimaging results are consistent with these findings insofar as dieters demonstrated greater similarity in the neural representation of food cues presented with and without calorie information when compared to non-dieters. One interpretation of this finding is that the exposure to health-relevant information was sufficient to temporarily motivate similar attention to the healthiness of food cues that dieters spontaneously represent.

Attending to calories may be one strategy by which dieters manage their weight. Extending this work to overweight or obese populations might reveal whether changes in food representation is associated with successful weight management. Relative to non-dieters and normal weight consumers, dieters and overweight consumers more frequently attend to the fat and sugar content of foods. Moreover, weight loss programs tend to increase the number of categories by which consumers evaluate foods (e.g., high calorie, likely to lead to weight loss, fills you up) [5]. Whether these naturally-occurring and programmatic strategies for evaluating foods successfully change consumers’ relationships with food in a way that contributes to weight management remains a question of interest. The best route to behavior change might incorporate a multi-step process that maximizes a person’s motivation to integrate calorie information into a food decision along with actual calorie information. In fact, though non-dieters’ behavior is typically less influenced by the presence of calories, when they are made aware of recommended daily calories—motivating their attention to this information—they reduce their eating behavior in a manner similar to dieters [7]. Adding health-related information, like the calorie content, to food labels might facilitate healthier choices, particularly for those individuals who are not independently motivated to consider this aspect of their food choices. Perhaps policy changes that combine calorie-labeled foods with increased public education about the importance of considering food calories will be the most effective at eliciting large-scale shifts in food choices.

## Supporting information

S1 Fig Activation in the OFC for NO CALORIES > CALORIES. **(A)** Compared to food images presented without calories, those presented with calories elicited marginally less activation in the OFC (6-mm sphere centered over -30, 33, -18), *B* = 0.13, 95% CI [-0.003, 0.26], *t*(40) = 1.92, *p* = 0.06. **(B)** This relationship did not differ for dieters and non-dieters, *B* = -0.07, 95% CI [-0.25, 0.11], *t*(40) = -0.75, *p* = 0.46.
(TIF)

S2 Fig Parametric modulation by food preferences in the OFC. (A) There was a stronger association between activation of the OFC (6-mm sphere centered over -30, 33, -18) and food preferences when evaluating food images with NO CALORIES than when evaluating food images with CALORIES in the reward system, *B* = 0.21, 95% CI [0.07, 0.35], *t*(40) = 2.82, *p* = 0.007. **(B)** This relationship did not differ for dieters and non-dieters, *B* = -0.07, 95% CI [-0.26, 0.11], *t*(40) = -0.75, *p* = 0.46.
(TIF)
