# Correction: Calorie information and dieting status modulate reward and control activation during the evaluation of food images

**DOI:** 10.1371/journal.pone.0226319

**Published:** 2019-12-05

**Authors:** Andrea L. Courtney, Emma K. PeConga, Dylan D. Wagner, Kristina M. Rapuano

Following the publication of this paper, the authors discovered that the description of the task presented in the in Materials & Methods omits an aspect of the task design. The following sentence should appear as the second sentence beneath Experimental design and procedure subheading of the Materials & Methods: These calories were slightly under- or over-reported (deviation of +/- 15% of total calories) for 2/3 of trials and were accurately reported for the remaining 1/3 of trials, and all were rounded to the nearest tens place.

The following corrections are required in accordance with this update:

The word “accurate” incorrectly appears in the final sentence beneath the Stimuli subheading of the Materials & Methods. The correct sentence is: The same images were displayed twice—once with the image number and once with calorie information (M = 396.5, SD = 240.2).

The Fig 1 legend incorrectly includes the word “accurate.” Please see the complete, correct Fig 1 and Fig 1 legend here.

**Fig 1:**
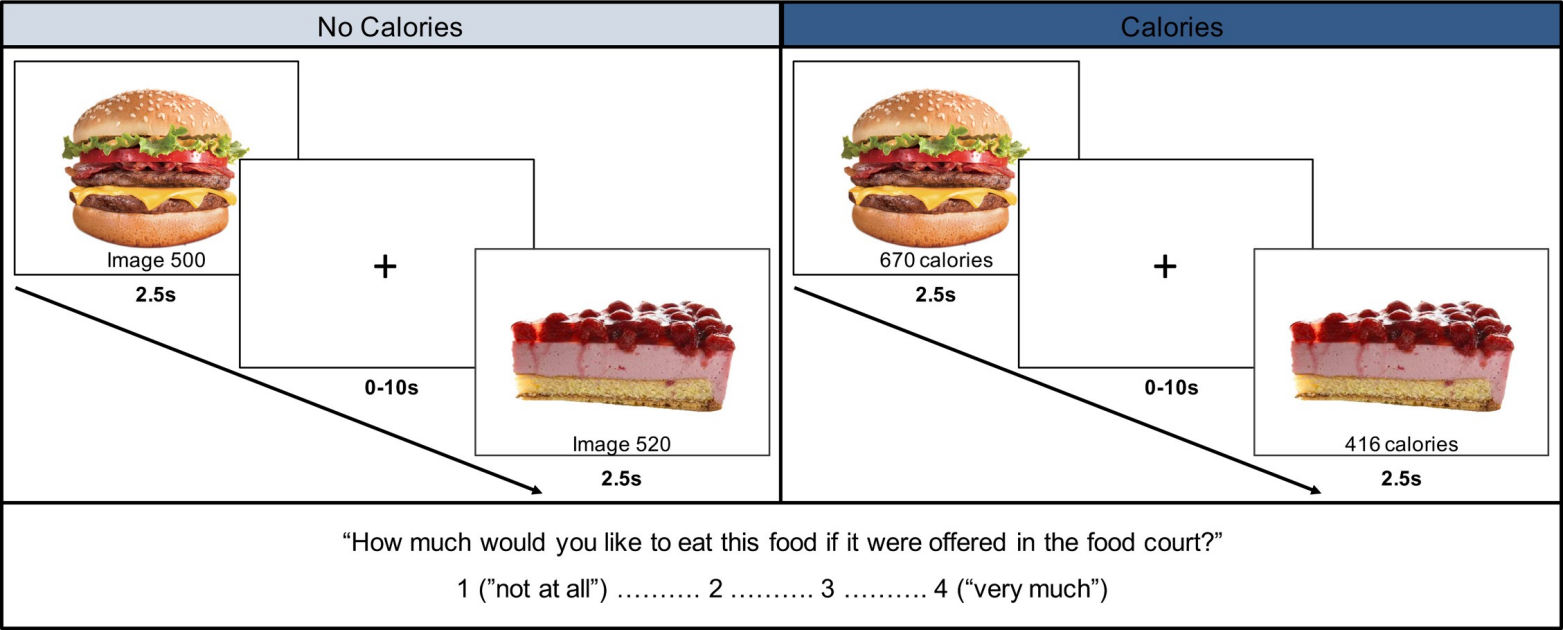
Representation of the food evaluation paradigm. 180 food images were presented (2.5s trial, 0-10s ITI) under two conditions: without calorie information and with calorie information. At the beginning of each run, participants were instructed to evaluate how much they would like to eat the food presented on each trial if they saw it in the campus dining hall (1 = “not at all”– 4 = “very much”).

In the Material & Methods, the final sentence of the second paragraph beneath the Experimental design and procedure subheading is incorrect. The correct sentence is: To obtain true estimates of food calories, rather than relying on participants’ memory for the previously presented calorie information, we informed participants that the calories they had observed during the task may have been altered for the study (and could be inaccurate) and should be disregarded when making their estimates.
