# Supplementary figures and images for: Calorie information and dieting status modulate reward and control activation during the evaluation of food images
Source: PLoS One. 2018 Nov 2;13(11):e0204744. doi: 10.1371/journal.pone.0204744 (PMC6214650; doi:10.1371/journal.pone.0204744)

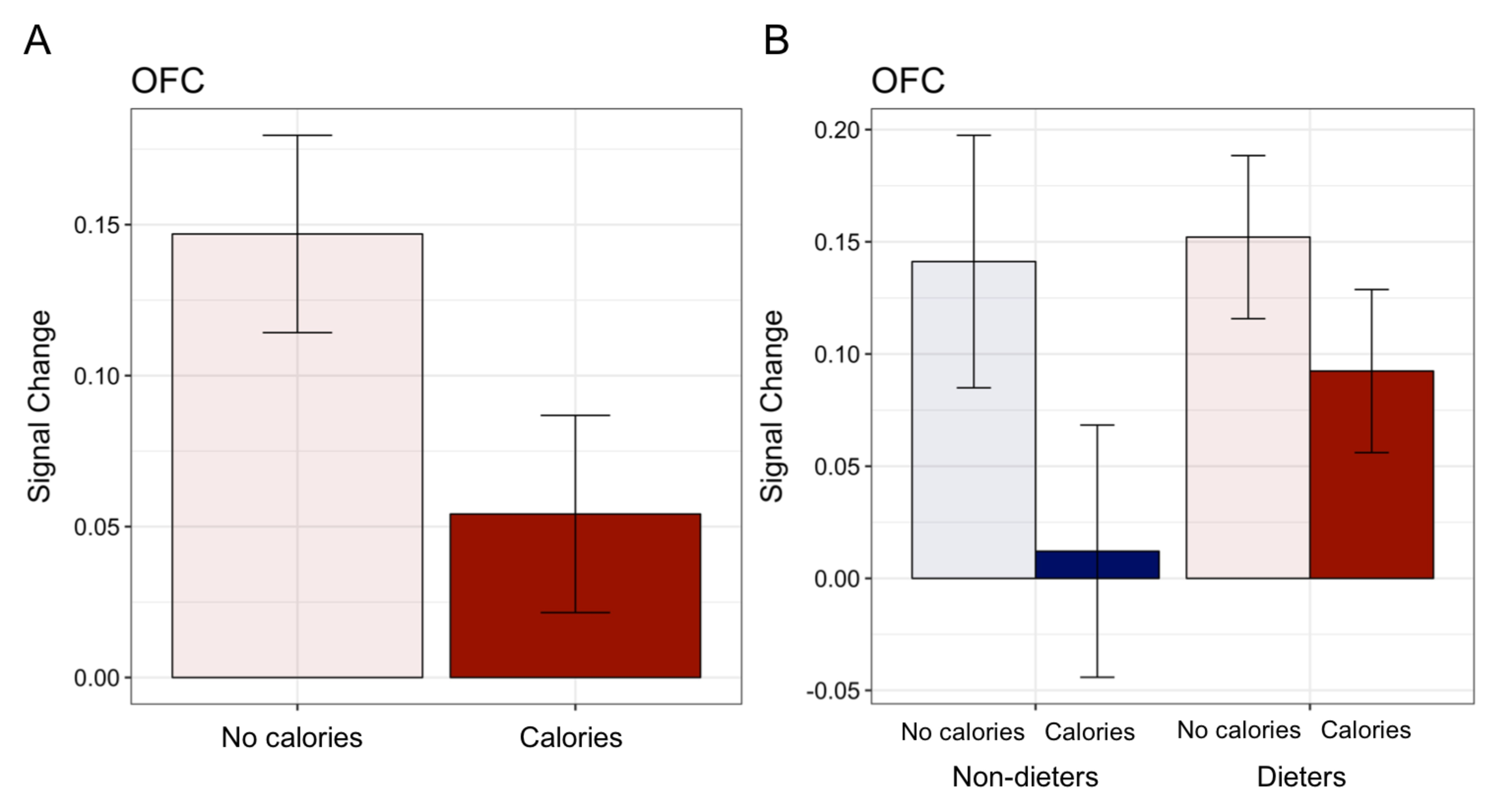

Supplement: S1 Fig — (A) Compared to food images presented without calories, those presented with calories elicited marginally less activation in the OFC (6-mm sphere centered over -30, 33, -18), B = 0.13, 95% CI [-0.003, 0.26], t(40) = 1.92, p = 0.06. (B) This relationship did not differ for dieters and non-dieters, B = -0.07, 95% CI [-0.25, 0.11], t(40) = -0.75, p = 0.46. (TIF) [file pone.0204744.s001.tif]

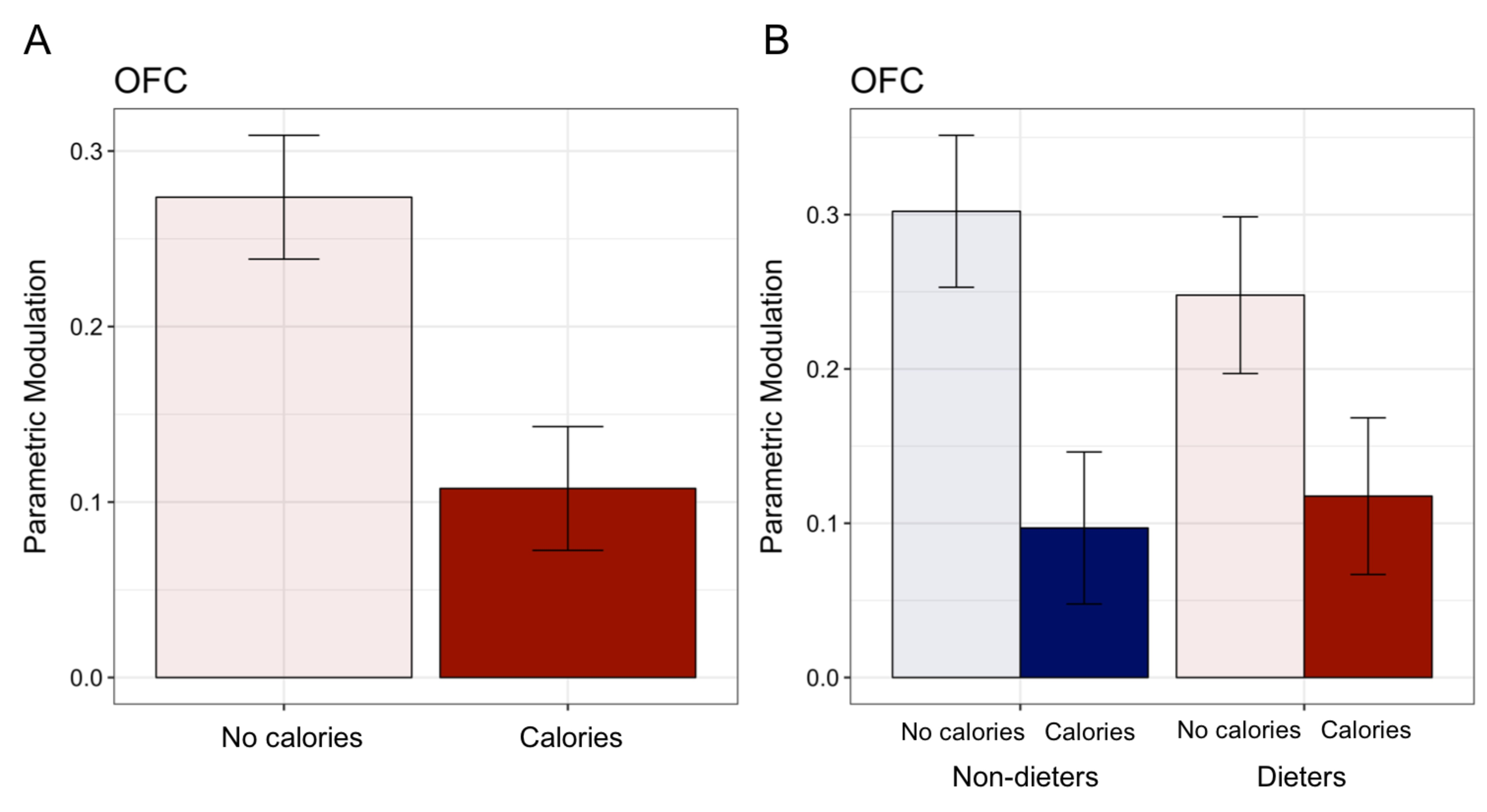

Supplement: S2 Fig — (A) There was a stronger association between activation of the OFC (6-mm sphere centered over -30, 33, -18) and food preferences when evaluating food images with NO CALORIES than when evaluating food images with CALORIES in the reward system, B = 0.21, 95% CI [0.07, 0.35], t(40) = 2.82, p = 0.007. (B) This relationship did not differ for dieters and non-dieters, B = -0.07, 95% CI [-0.26, 0.11], t(40) = -0.75, p = 0.46. (TIF) [file pone.0204744.s002.tif]
